# Accounting for filter bandwidth improves the quantitative accuracy of bioluminescence tomography

**DOI:** 10.1117/1.JBO.20.9.096001

**Published:** 2015-09-01

**Authors:** Shelley L. Taylor, Suzannah K. G. Mason, Sophie L. Glinton, Mark Cobbold, Hamid Dehghani

**Affiliations:** a University of Birmingham, PSIBS Doctoral Training Centre, Edgbaston, Birmingham B15 2TT, United Kingdom; b University of Birmingham, School of Computer Science, Edgbaston, Birmingham B15 2TT, United Kingdom; c University of Birmingham, College of Medical and Dental Sciences, School of Immunity and Infection, Edgbaston, Birmingham B15 2TT, United Kingdom

**Keywords:** bioluminescence imaging, bioluminescence tomography, image reconstruction

## Abstract

Bioluminescence imaging is a noninvasive technique whereby surface weighted images of luminescent probes within animals are used to characterize cell count and function. Traditionally, data are collected over the entire emission spectrum of the source using no filters and are used to evaluate cell count/function over the entire spectrum. Alternatively, multispectral data over several wavelengths can be incorporated to perform tomographic reconstruction of source location and intensity. However, bandpass filters used for multispectral data acquisition have a specific bandwidth, which is ignored in the reconstruction. In this work, ignoring the bandwidth is shown to introduce a dependence of the recovered source intensity on the bandwidth of the filters. A method of accounting for the bandwidth of filters used during multispectral data acquisition is presented and its efficacy in increasing the quantitative accuracy of bioluminescence tomography is demonstrated through simulation and experiment. It is demonstrated that while using filters with a large bandwidth can dramatically decrease the data acquisition time, if not accounted for, errors of up to 200% in quantitative accuracy are introduced in two-dimensional planar imaging, even after normalization. For tomographic imaging, the use of this method to account for filter bandwidth dramatically improves the quantitative accuracy.

## 1 Introduction

Bioluminescence imaging (BLI) is a widely used tool in preclinical research to image and monitor ongoing biological processes using small animal models. The technique involves labeling a cell type of interest with a bioluminescent reporter gene, which emits light when a substrate is introduced. Light (typically in the red/near-infrared region) which reaches the surface of the imaging subject is captured, enabling conclusions to be drawn about the location and dynamics of the labeled cells. The technique is widely used in cancer research, enabling tumor growth and the efficacy of novel treatments to be monitored;^1^^–^^3^ the migration and localization of stem cells,^4^^,^^5^ immune cells,^6^^,^^7^ bacteria,^8^^,^^9^ and viruses^8^^,^^10^^,^^11^ have also been investigated.

An extension of BLI is bioluminescence tomography (BLT), which provides three-dimensional (3-D) spatially resolved volumetric information about source location and intensity. Typically, BLI images are captured at multiple wavelengths through the use of bandpass filters within the range of the spectral emission of the source. This multispectral data are then used by the reconstruction algorithm to recover the strength and distribution of the internal source.^12^^–^^14^

The use of multiple wavelengths (ranging from 3 to 5 wavelengths within the emission spectrum of the source) for tomographic imaging has been shown to be important as the BLT reconstruction problem is inherently ill-posed, resulting in nonunique solutions for the source recovery.^15^ By imaging at multiple wavelengths and exploiting the spectral attenuation of the underlying tissue and the spectral emission of the source, the set of possible solutions is further constrained, which improves the quality of the image reconstruction.

When performing preclinical BLI and BLT, the data acquisition time must be as short as possible; although the underlying physiology of the tissue being imaged is stable over the imaging timescale, there is a short window of time (∼10 to 20 min),^16^ where the maximum emission intensity of firefly luciferase (fluc, a widely used bioluminescent reporter gene) occurs, and the emission is stable. This window varies depending on the animal model and type of luciferase used,^16^^,^^17^ so the emission characteristics must be determined in each specific experiment before data can be taken.^16^ As multispectral images must be taken within this time window, it is imperative to minimize the acquisition time in order to maximize the number of images which can be taken in one imaging session. Additionally, reducing the acquisition time will enable a larger number of animals to be imaged in one session, while minimizing the time an animal is anaesthetized.

The data acquisition time is dependent on multiple factors, including the signal strength and the attenuation of the underlying tissue, as well as the system and devices used for data capture. Given that the signal strength and tissue attenuation are dependent on the unknown underlying physiology, and assuming a highly sensitive device such as a cooled charge-coupled device (CCD) for data capture, one method to reduce the acquisition time while maximizing the detected signal is to perform the multispectral imaging using wide bandwidth filters with high transmission.

**Fig. 1:**
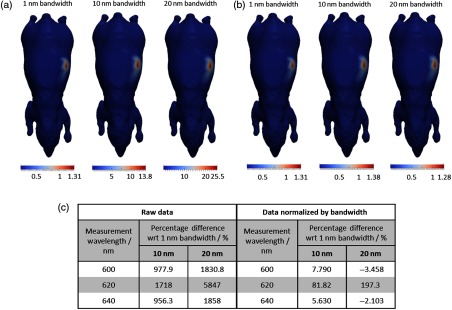
(a) Forward data calculated at 600 nm for a heterogeneous mouse model with a bioluminescent pancreas, at three bandwidths, thresholded at 12.5% of the maximum value for visualization. The same distribution is seen but the intensity varies with bandwidth. (b) Forward data at 600 nm normalized by bandwidth, thresholded at 12.5% for visualization. (c) Percentage difference in total intensity (thresholded at 50% of maximum value) of forward data between bandwidths of 10 and 20 nm and 1 nm. Shown for raw data and data that have been normalized by bandwidth.

To demonstrate the effect of increasing the bandwidth of filters used on the measured signal, a simple case has been simulated using a heterogeneous mouse model (detailed later) with a bioluminescently labeled pancreas. Surface fluence data are calculated using NIRFAST^18^ for three separate cases, assuming a bandwidth of 1, 10, and 20 nm. Figure 1(a) shows the planar bioluminescence at 600 nm using different bandwidth filters demonstrating that the surface distribution of the light is qualitatively the same in all cases, but the intensity is higher for the wider bandwidths. As is evident in Fig. 1(c), the signal at 600 nm from a bandwidth of 10 nm is ∼980% larger than that from 1-nm bandwidth. This suggests that a much shorter acquisition time (factor of ∼9.8×) is required in order to obtain the same measurement intensity. When using a bandwidth of 20 nm, this reduction in acquisition time is ∼18×. This highlights the importance of using wider bandwidth filters for preclinical studies.

Using multispectral data for 3-D tomographic image recovery exploits the spectral properties of the domain being imaged and is considered to improve accuracy by reducing the nonunique nature of the BLT problem. However, the bandwidth of the filters used for image acquisition is currently not considered within the reconstruction algorithm, with the data assumed to be collected at the central wavelength of the filters only.

For a given filter of known bandwidth, data are collected over a range of wavelengths. For example, for a filter with a central wavelength of 600 nm and a bandwidth of 20 nm, data are collected for all wavelengths ranging from 590 to 610 nm. Ignoring this and assuming that all data comes from the central filter wavelength leads to a model-data mismatch for model-based image recovery. This is because the range of wavelengths transmitted through the filter, and therefore, the spectral properties of both tissue attenuation and bioluminescent source emission within the bandwidth of the filter are not considered. This results in a loss of accuracy in the reconstruction.

For tomographic applications using BLT, if the reconstruction algorithm used is modified to account for the bandwidth of each filter, the quantitative accuracy of the spatially resolved source is expected to improve. In this work, the algorithms used in model-based image recovery for BLT are modified from a single wavelength [narrow bandwidth (NBW)], to account for the bandwidth of the filters used resulting in a wide bandwidth (WBW) model. The effect of using this WBW model on the recovered source accuracy is demonstrated and assessed using both simulation data from a complex mouse model and experimental data using a mouse phantom.

We investigate the reduction of the image acquisition time in a recently developed imaging system, a multimodal BLT, and diffuse optical tomography (BLDOT) system.^19^ The filters currently used in the BLDOT system are 10-nm bandwidth band-pass filters with central wavelengths ranging from 600 to 640 nm, which result in exposure times of ∼2 to 3 min/wavelength. Increasing the bandwidth of the filters to ∼20  nm is investigated and shown to improve the acquisition time without any detriment to the quantitative accuracy of the recovered bioluminescent source from a mouse phantom.

## 2 Theory

When performing bioluminescence tomographic reconstructions, surface-weighted measurements are used along with reconstruction algorithms to recover the internal bioluminescent source.^15^ Given a known source distribution, x, and a model of light propagation, J, the boundary measurements, b, are given by a linear relationship,

Jx=b. (1)

Therefore, to recover the unknown source distribution, x, from a set of known boundary measurements, b,

$$x=J^{-1}b.$$ (2)

The sensitivity matrix, or Jacobian J, is created from a combination of Jacobians calculated for each of the n measurement wavelengths,

$$J=[J_{\lambda_{1}^{*}};J_{\lambda_{2}^{*}};\ldots J_{\lambda_{n}^{*}}],$$ (3)

where $\lambda_{i}^{*}$ is the measurement at wavelength i. The measurement matrix b is, therefore, also a combination of measurements taken at each measurement wavelength.

$$b=[b_{\lambda_{1}^{*}};b_{\lambda_{2}^{*}};\ldots b_{\lambda_{n}^{*}}].$$ (4)

Since the measurements, $b_{\lambda_{i}^{*}}$, are usually assumed to be from a single wavelength (the central filter wavelength, $\lambda_{i}^{*}$), the corresponding Jacobians are also calculated according to this assumption; these Jacobians will be referred to as the NBW Jacobians. However, typically for any given system, bandpass filters are used for data acquisition, and the measurements are comprised of signals from all wavelengths which are transmitted through the filter, governed by its bandwidth. Therefore, assuming a top-hat bandpass filter, measurements at each wavelength are instead defined as

$${\overset{˜}{b}}_{\lambda_{i}^{*}}=\overset{\lambda_{i}^{*}+\frac{w}{2}}{\underset{\lambda_{j}=\lambda_{i}^{*}-\frac{w}{2}}{\sum }}b_{\lambda_{j}},$$ (5)

where $b_{\lambda_{j}}$ is the measurement contribution by each wavelength transmitted through the filter with bandwidth w. By the same definition, the Jacobian to be considered for each measurement wavelength is defined as

$${\overset{˜}{J}}_{\lambda_{i}^{*}}=\overset{\lambda_{i}^{*}+\frac{w}{2}}{\underset{\lambda_{j}=\lambda_{i}^{*}-\frac{w}{2}}{\sum }}J_{\lambda_{j}},$$ (6)

where $J_{\lambda_{j}}$ is the Jacobian matrix calculated for each wavelength transmitted through the filter. Using the definitions above, Eq. (1) now becomes

$$\overset{˜}{J}x=\overset{˜}{b},$$ (7)

where $\overset{˜}{J}$ is the modified Jacobian accounting for the bandwidth of the filters used and will be referred to as the WBW Jacobian.

In order to evaluate the effects of considering the filter bandwidth for image recovery, both the NBW and WBW Jacobians described above are used in this study, together with a compressive sensing conjugate gradient (CSCG) based method^20^ for image reconstruction. The CSCG algorithm assumes a sparse source distribution, as is typically the case when studying the growth and kinetics of localized cancerous tumors, which has been shown to reduce the inherent ill-posed nature of BLT reconstructions.^20^

## 3 Methods

### 3.1 Simulation Using a Complex Mouse Model

**Fig. 2:**
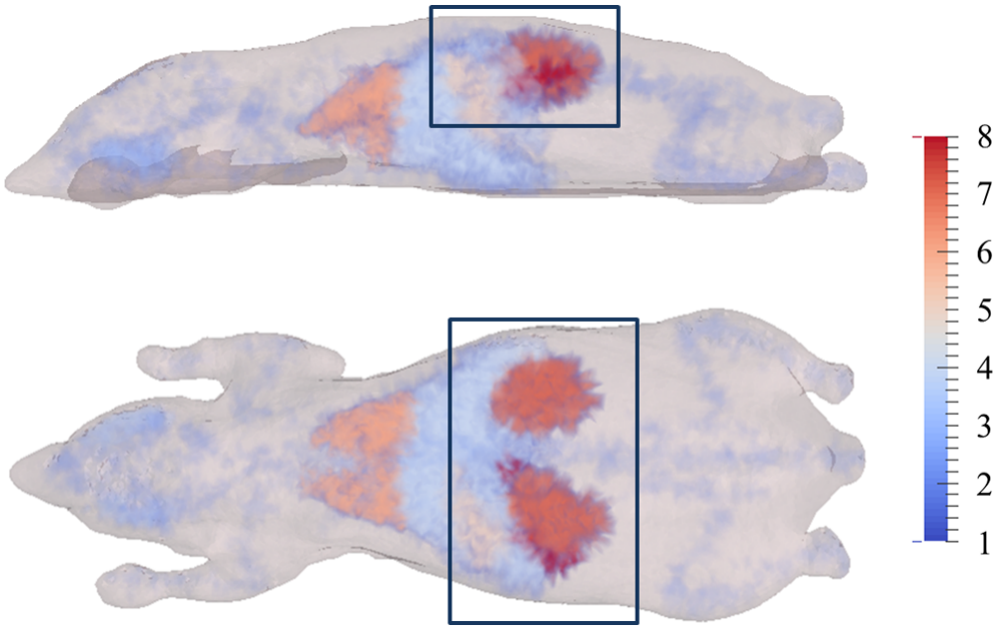
Mouse mesh used in the simulations. The color bar indicates the regions of the mesh, as detailed in Table 1. Region 8 is the pancreas, which was made bioluminescent. 451 detectors were positioned uniformly across the surface of the mesh in the boxed area.

**Fig. 3:**
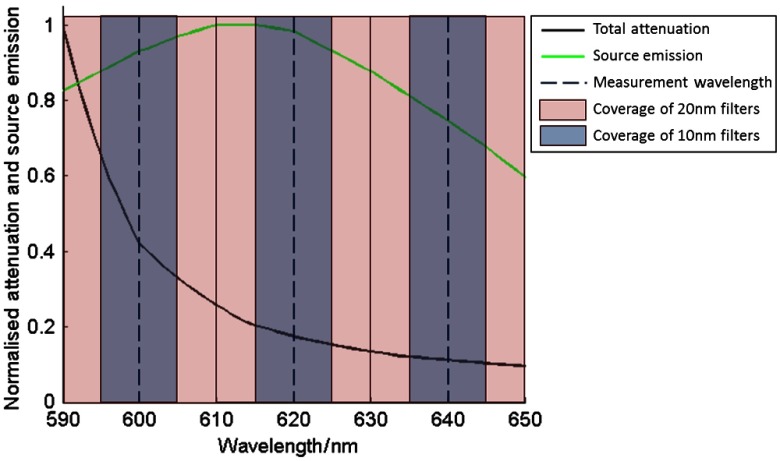
Total attenuation of tissue and source (fluc) emission, both normalized by the maximum value, as a function of wavelength for the computational model, across the wavelength range used in this study. Dotted vertical lines indicate the measurement wavelengths used, and the sampling coverage of 1-nm bandwidth data. The blue and red shaded areas indicate the coverage of the 10 nm and 20 nm data, respectively. It can be seen that the 20-nm filters collect data over the entire spectral range used in this study.

A 3-D heterogeneous mouse model (the Digimouse atlas^21^) with a bioluminescent pancreas (Firefly Luciferase, fluc) was used for simulation studies (Fig. 2). Tissue properties of the eight regions of the model are shown in Table 1^22^ and the optical properties for each wavelength used were calculated using appropriate extinction coefficients (http://omlc.org/spectra/). A total of 451 detectors were modeled over the surface of the animal above the pancreas and simulated data were calculated using NIRFAST^18^ at three measurement wavelengths, $\lambda^{*}=600,620,640\;\mathrm{nm}$, and eight different bandwidths (1, 2, 4, 6, 8, 10, 16, and 20 nm). The source emission spectrum and tissue attenuation over the wavelength range simulated are shown in Fig. 3, along with the spectral coverage of the 1-, 10-, and 20-nm filters. Reconstructions based on the noise-free data were performed using the CSCG algorithm using both NBW and WBW Jacobians.

**Table 1:** Optical properties of the mouse mesh. Reproduced from Ref. 22.

Region	Tissue	Total hemoglobin, mM	Oxygen saturation, %	Water concentration, %	Scatter amplitude	Scatter power
1	Adipose	0.0033	70	50	0.98	0.53
2	Bone	0.0049	80	15	1.4	1.47
3	Muscle	0.07	80	50	0.14	2.82
4	Stomach	0.01	70	80	0.97	0.97
5	Lungs	0.15	85	85	1.7	0.53
6	Kidneys	0.0056	75	80	1.23	1.51
7	Liver	0.3	75	70	0.45	1.05
8	Pancreas	0.3	75	70	0.45	1.05

Using the recovered images of internal source distribution, the center-of-mass (COM), equivalent radius (radius of a sphere of the same volume as the recovered source, mm) and the total intensity of the source were calculated. This enabled quantitative evaluation of the results for each bandwidth for all models. Only data with intensities above a threshold of 2% of the maximum value were considered here. This threshold was empirically chosen and is low due to the very low intensity noise in the simulated data.

### 3.2 Experimental Data Using a Mouse Phantom

**Fig. 4:**
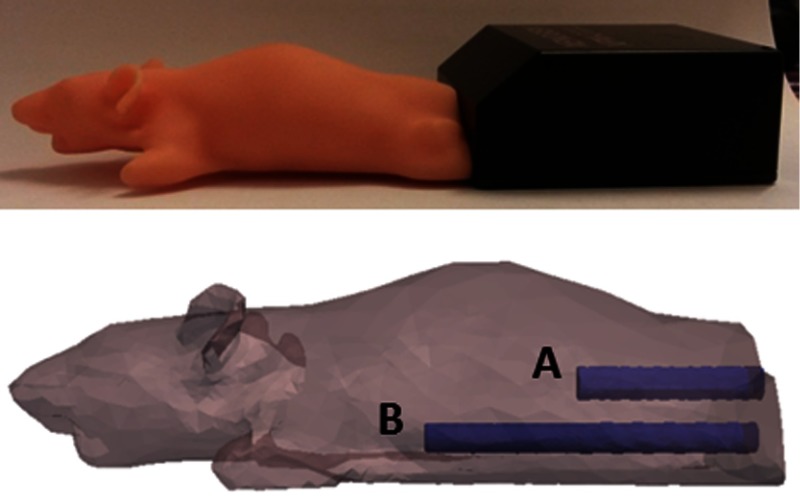
XPM mouse-shaped phantom. Position of the two sources and internal rods are indicated in the lower image, obtained using microCT data.

A plastic mouse phantom (XPM2, Perkin Elmer, Fig. 4), with two internal light sources, was imaged using the BLDOT system. Images were taken at $\lambda^{*}=600$, 620, and 640 nm with 10-nm bandwidth filters (Thorlabs Inc.) and at $\lambda^{*}=600$, 623, and 643 nm, where each filter had a different bandwidth: 22, 32, and 34 nm, respectively (Semrock Inc.). The emission characteristics of the sources within the XPM2 phantom have been previously characterized and the spectral optical properties have been experimentally determined^23^ and are accounted for within the reconstruction. Surface measurements were calibrated using a free-space model,^24^ which uses the bioluminescence images taken during the experiment along with information about the dimensions of the imaging system, to model the transport of light through the space between the surface of the imaging subject and the CCD. This enables accurate surface measurements to be calculated from raw CCD data.

The COM and equivalent radius of the reconstructed source (both using a data threshold of 10% chosen due to systematic noise present), and the total intensity were calculated in order to provide quantitative comparisons between reconstructions with NBW and WBW Jacobians.

## 4 Results and Discussion

### 4.1 Simulation

#### 4.1.1 Effect of filter bandwidth

Raw measurement data are shown for a range of bandwidths (1, 10, and 20 nm) in Fig. 1(a). The modeled surface intensity is greater for data simulated at larger bandwidths for a given exposure time, as is expected due to the increase in the total light intensity transmitted through the filter, arising from the larger number of transmitted wavelengths. The percentage difference (PD) between the maximum measured intensity at 10 and 20 nm bandwidths and 1-nm bandwidth, calculated using the equation: $\mathrm{PD}=(I_{10\;\mathrm{nm}}-I_{1\;\mathrm{nm}}/I_{1\;\mathrm{nm}})\times 100$, shows the extent to which the signal varies [Fig. 1(c)]. Considering only the raw data, the maximum PD for 10-nm data with respect to 1-nm data is 1718% while that for 20-nm data is 5847%. The maximum difference is seen at 620 nm in all cases, as the source emission is close to the peak intensity of fluc (612 nm)^25^ and the tissue attenuation is low (Fig. 3).

A possible method to remove this intensity dependence is to normalize the measurement data by the appropriate bandwidth, i.e., data taken using a bandwidth of 10 nm is divided by 10, etc. This results in intensity distributions with similar maximum intensities at each bandwidth [Fig. 1(b)]. However, when calculating the PD between data simulated at the two larger bandwidths (10 and 20 nm) and the smaller bandwidth (1 nm), there still exists a variation of up to 82% for 10-nm data and 197% for 20-nm data, as compared to 1-nm data. The PD for 20-nm bandwidth data at measurement wavelengths of 600 and 640 nm is negative, indicating a lower normalized signal as compared to the 1-nm data. Due to the large intensity variation still present, normalization is not an appropriate method to use to account for the bandwidth of filters used.

**Fig. 5:**
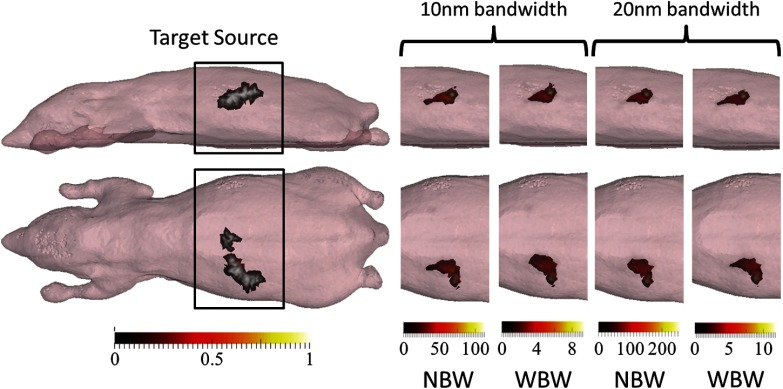
Side and top view of target and reconstructed sources. Reconstructions shown were performed on data simulated with 10 nm and 20 nm filters and with narrow bandwidth (NBW) and wide bandwidth (WBW) Jacobians. The reconstructions are qualitatively similar for both Jacobian types and there is no effect of bandwidth on the reconstructed source. Color bar units: arbitrary intensity units.

In the case of BLT, the target source and the sources recovered from simulated data at 10 and 20 nm bandwidths are shown in Fig. 5. There are qualitatively no differences between the reconstructed source distributions at the two bandwidths when considering the NBW model.

**Fig. 6:**
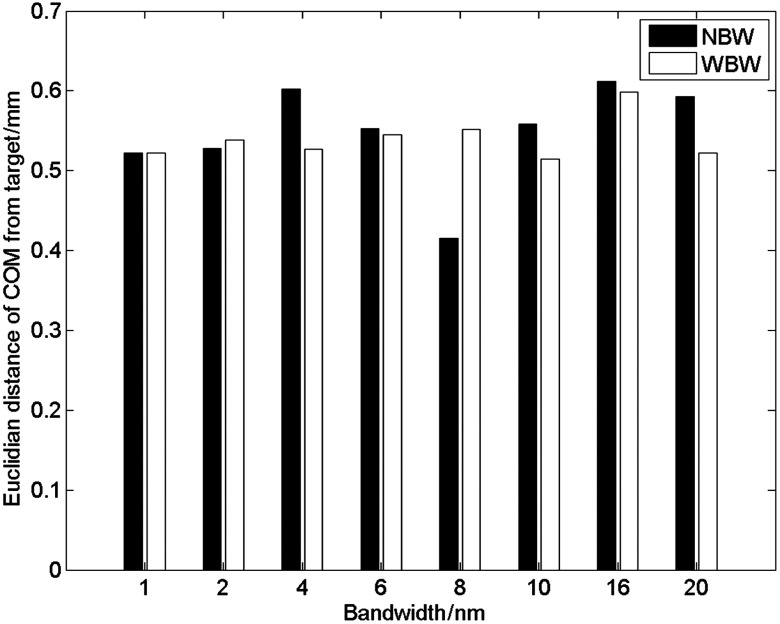
Error in center-of-mass of sources reconstructed using NBW and WBW Jacobians at a range of bandwidths.

**Fig. 7:**
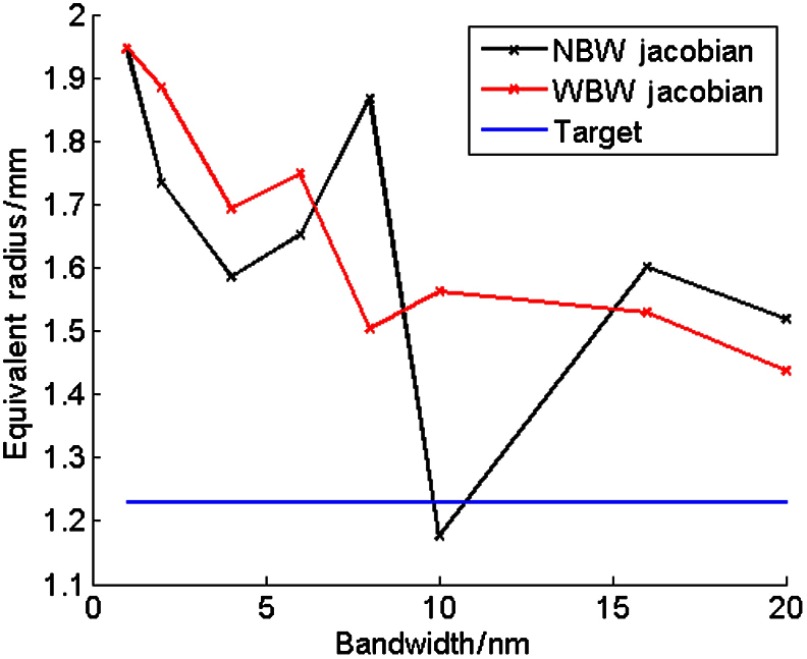
Equivalent radius (mm) of sources reconstructed using NBW and WBW Jacobians at a range of bandwidths, with the target source value (1.23 mm) displayed for comparison.

The COM error (Fig. 6) of the reconstructed sources, i.e., the Euclidian distance between the COM of the reconstructed source and the target source, using the NBW model remained constant to within 0.2 mm over the range of bandwidths simulated. Some variation is observed in the equivalent radius (maximum variation, 0.77 mm, Fig. 7), however, this does not follow a trend with bandwidth, and is a small variation, suggesting that bandwidth has little effect on the recovered source size and position. The equivalent radius further demonstrates the observed similarity between the recovered sources at 10- and 20-nm bandwidths (Fig. 5), which have equivalent radii of 1.18 and 1.52 mm, respectively.

**Fig. 8:**
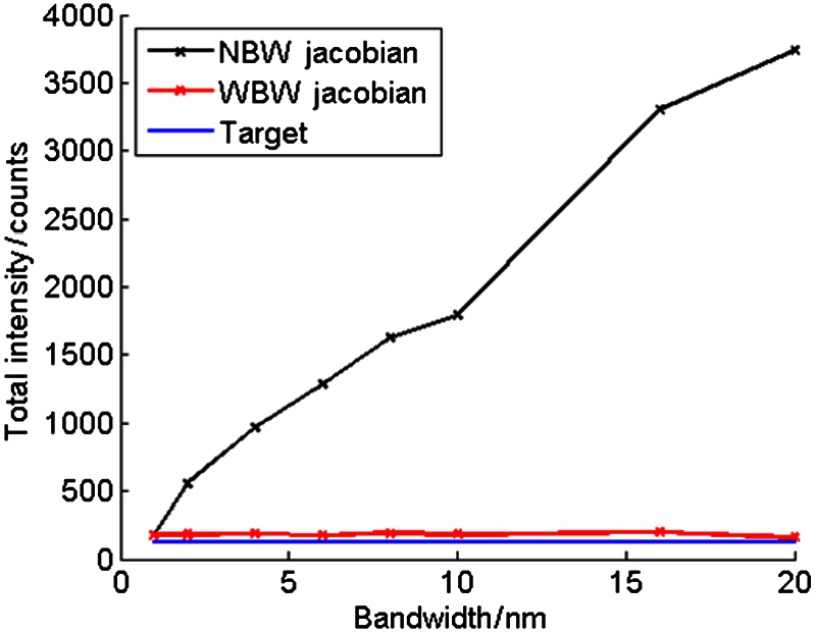
Total intensity of reconstructed sources for NBW and WBW Jacobian reconstructions, with the target value (124) shown. The increased quantitative accuracy of the WBW reconstructions is demonstrated. The NBW reconstruction intensity increases with bandwidth, increasing the difference between the target intensity and reconstructed intensity. Reconstructing with the WBW Jacobian removes this dependence.

Variation in the total recovered source intensity with bandwidth is apparent when using the NBW Jacobian (Fig. 8). As bandwidth increases, the discrepancy between the total intensity of the target and the reconstructed sources increases dramatically, resulting in the intensity of the source recovered using the 20-nm data being ∼20× higher than that using 1-nm data. This is due to an increase in the model-data mismatch as the bandwidth increases when using the NBW model. At higher bandwidths, it becomes less appropriate to approximate the data as being collected at the single, central wavelength of the filter, as data taken using larger bandwidth filters is affected to a greater extent by the spectrally varying optical properties of tissue and source emission.

This result demonstrates that changing the bandwidth of filters does affect the tomographic reconstructions when the NBW Jacobian model is used to reconstruct. There is very little variation in the position and size of the recovered source, but the intensity of the reconstruction is greatly affected. Normalization is not considered a viable method of accounting for the bandwidth of the filters in BLT as the spectral variation of the underlying tissue attenuation and the source emission are not accounted for. If BLT were to be performed using normalized data, the model-data mismatch in the reconstruction algorithm would still be present, resulting in inaccurate source reconstructions.

The sources were also recovered using the modified WBW Jacobian (detailed in Sec. 2) to take into account the effects of the spectrally varying data, as discussed next.

#### 4.1.2 Effect of Jacobian type

The recovered source distribution using NBW and WBW Jacobians at bandwidths of 10 and 20 nm is shown in Fig. 5. The position and distribution of the recovered sources using the two Jacobian types are qualitatively very similar, supported by the error in COM (Fig. 6) and the equivalent radius (Fig. 7).

The error in COM of the reconstructions using the two different Jacobian models shows little variation at all simulated bandwidths (the largest variation between the two Jacobian types is approximately 0.14 mm, Fig. 6). The equivalent radius decreases with bandwidth in WBW reconstructions in contrast to the random variation observed for NBW reconstructions (Fig. 7), but this variation is only 0.51 mm (0.77 mm for NBW equivalent radii). The two larger bandwidth filters have smaller equivalent radii, which are closer to the target value of 1.23 mm, for WBW compared to NBW as the model-data mismatch has been successfully accounted for.

The real advantage of using the WBW Jacobian model for image reconstruction is demonstrated when total intensity is considered (Fig. 8). As described above, the total intensity of the NBW reconstructions increases dramatically as bandwidth is increased due to the model-data mismatch in the reconstruction algorithm becoming greater at larger bandwidths. This variation in intensity is removed by using the WBW model. The intensity of the source recovered from the 20-nm data is now 0.88× that of the 1-nm data (compared to 20× for the NBW model), with the intensity of all reconstructions within 58% of the target value (as opposed to 2918% for the NBW model).

The time taken to perform the reconstruction using the WBW model increases with the bandwidth of the filter, as compared to the constant reconstruction time when using the NBW model. The computation time for NBW and WBW reconstructions as a fraction of the time taken to reconstruct 1-nm data is shown in Table 2 with an evident increase in reconstruction time with increasing bandwidth for WBW reconstructions. For reference, for a workstation running on 64-bit Windows (Windows 7 Enterprise) with 16 GB RAM, using an Intel® Core™ i7-3770 CPU at 3.40 GHz, using MATLAB R2013a, the NBW and WBW reconstructions for 1 nm took ∼2960  s. This increase in processing time is due to the nature of the WBW model, which calculates Jacobians at each transmitted wavelength and combines them, whereas the NBW model calculates one Jacobian only at the central filter wavelength regardless of the bandwidth of the filter. Although the processing time is increased, the increase in quantitative accuracy of the recovered sources demonstrates that using the WBW model will advance BLI and BLT toward becoming truly quantitative imaging techniques.

**Table 2:** Reconstruction time for simulation data relative to the time taken to reconstruct data at 1-nm bandwidth. The increase in time to reconstruct wider bandwidth data using the wide bandwidth (WBW) method is shown.

	Bandwidth, nm	Reconstruction time as a fraction of 1-nm time	
		NBW	WBW
Simulation	1	1	1
	2	1.19	1.14
	4	1.01	1.36
	6	1.14	1.67
	8	1.04	1.99
	10	1.00	1.88
	16	1.12	2.80
	20	1.04	2.94

Note: NBW, narrow bandwidth.

### 4.2 Experiment

**Fig. 9:**
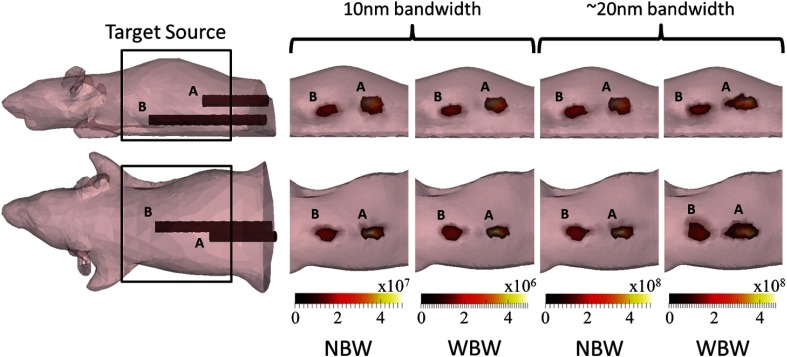
Target source locations for the XPM phantom (at the end of the rods shown in the images) and top and side views of XPM source reconstructions, thresholded at 20% of the maximum value, using the narrower (10 nm) and wider (∼20  nm) bandwidth filters and NBW and WBW Jacobians. Barely any difference is observed between the reconstructions with different bandwidth data and the two Jacobian types. Color bar units: intensity/counts $s^{-1}$.

Sources recovered from experimental data of the XPM2 mouse-shaped phantom with the two internal sources (Fig. 4) illuminated, taken using 10 nm and wider (∼20  nm) bandwidth filters and using both NBW and WBW Jacobian models, are shown in Fig. 9. Little difference is observed in terms of position and appearance of the recovered source, when comparing both bandwidths and Jacobian models. This suggests qualitatively that bandwidth has no effect on the source reconstructions, as with simulation, and as the total imaging time is decreased from 405 to 80 s, it is experimentally advantageous to use wider bandwidth filters. This will enable the number of images taken during the time at which the emission of fluc is stable to be maximized.

The error in COM of the reconstructions (Table 3) is very similar for both NBW and WBW Jacobian models at both bandwidths. Note the larger error for source B, which is primarily due to the greater depth of the source within the phantom as compared to source A. The equivalent radius is also very similar for both NBW and WBW Jacobian models (Table 3). This, along with qualitative observations of the reconstructions, supports the findings of the simulations that in terms of the size and position of the recovered source, there are very few differences between reconstructions using data at different bandwidths when using either the NBW or WBW Jacobian models. However, as with the simulations, the real advantage of using the WBW Jacobian model to reconstruct is clear when the intensity of the source reconstruction is considered.

**Table 3:** Quantitative analysis of XPM source reconstructions. The larger bandwidth filters used for imaging are referred to as having a 20-nm bandwidth in this table.

Bandwidth, nm	Jacobian type	Center-of-mass error, mm		Equivalent radius, mm		Total intensity	
		A	B	A	B	A	B
10	NBW	1.62	3.58	0.65	1.60	$4.89\times 10^{8}$	$2.88\times 10^{8}$
20	NBW	1.71	3.63	0.65	1.53	$2.33\times 10^{9}$	$1.44\times 10^{9}$
10	WBW	1.59	3.84	0.42	1.47	$4.25\times 10^{7}$	$2.50\times 10^{7}$
20	WBW	2.03	4.28	1.13	1.89	$6.20\times 10^{7}$	$3.79\times 10^{7}$

The total reconstruction intensity of data taken with the wider bandwidth filters using the NBW Jacobian is approximately five times greater than that using narrow bandwidth data, for both sources (Table 3). There should be no difference in intensity between reconstructions from different bandwidth data as identical sources are being reconstructed in both cases.

When the WBW Jacobian model is used to reconstruct, the difference in intensity is dramatically decreased (Table 3). The reconstructed intensity using wider bandwidth data is just 1.5× that using narrower bandwidth data. Therefore, accounting for the bandwidth of filters in reconstructions removes the dependence of the intensity of reconstructions on the bandwidth of the filters used in imaging. However, as with the simulation study, the increased number of Jacobians, which are calculated during the reconstruction, results in an increase in the reconstruction time when using the WBW model as compared to the NBW model, with data taken using wider bandwidth filters taking the longest to reconstruct.

Experimental results support the simulation work: using the WBW Jacobian model to reconstruct dramatically increases the quantitative accuracy of reconstructions. There is qualitatively little effect on the source reconstructions, in terms of COM error and equivalent radius, but quantitatively, the difference between the total reconstruction intensity at different bandwidths is removed.

## 5 Conclusions

This work shows how changing the bandwidth of filters used for BLI data acquisition quantitatively affects the recovered source in BLT, both through simulation using a heterogeneous mouse model and experiment using a mouse phantom. The recovered source intensity was shown to have a strong dependence on the bandwidth of the filters used for data acquisition, demonstrating the importance of accounting for the bandwidth in the reconstruction algorithm when performing quantitative BLT.

A method of Jacobian modification to account for the filter bandwidth is presented, resulting in a WBW Jacobian model, where both the spectrally varying emission of the source as well as underlying tissue attenuation is considered. The distribution and intensity of the source recovered using the WBW Jacobian model is compared to that using the traditionally used NBW Jacobian model, which assumes that data are collected at a single wavelength (the central filter wavelength).

The source recovered from simulated data of a heterogeneous mouse model with a bioluminescent pancreas showed little variation in distribution and position with both bandwidth and Jacobian type. However, a linear dependence of recovered source intensity on bandwidth was found in NBW-based reconstructions. When the WBW Jacobian model was used to reconstruct, this intensity dependence was removed, resulting in quantitative information independent of the bandwidth of the filter. This demonstrates both the necessity to account for the filter bandwidth when performing quantitative BLT and the efficacy of the proposed WBW Jacobian.

Experimental data using a mouse phantom with two internal light sources supported the simulation findings. Little variation in the distribution and position of the recovered source was found when comparing sources recovered from data taken at different bandwidths and using both Jacobian types. There exists a variation in intensity with bandwidth when considering NBW-based reconstructions, with sources recovered from wider (∼20  nm) bandwidth data ~5× the intensity of those from data taken using 10-nm bandwidth filters. The WBW Jacobian model gives an intensity variation of ∼1.5× between the two bandwidths used. Therefore, the WBW Jacobian has been shown to effectively account for the bandwidth of the filters used in imaging, while reducing the data acquisition time by a factor of 5.

The efficacy of the WBW method in increasing the quantitative accuracy is demonstrated in this work. However, it should be noted that while the imaging time is decreased when using larger bandwidth filters, the postimaging processing time is increased using the WBW method (as shown in Table 2). This is due to the nature of the method, that Jacobians are calculated at each wavelength which is transmitted through the filter, therefore, the wider the bandwidth the longer the processing time. In order to do this, knowledge of the spectral transmission properties of the filters must be known, which is readily available from manufacturers. Despite the increase in processing time, the substantial advantages of using the WBW method will advance BLT toward becoming a truly quantitative imaging technique.

Accounting for the bandwidth of filters used for imaging has been shown to make a considerable difference to the quantitative accuracy of the recovered sources when performing BLT. Many preclinical studies use the bioluminescent signal as a way of quantifying cell proliferation, e.g., in the case of tumor growth^1^^,^^26^ or infection or disease progression.^27^^,^^28^ Therefore, providing a way of achieving quantitatively accurate intensity information about the source will enable accurate conclusions about the dynamics and size of the source to be drawn.

In preclinical cancer research, more specifically metastasis,^29^^,^^30^ the bioluminescent signal from small metastatic lesions is low.^1^ Therefore, in order to perform quantitative BLT to provide location and intensity information about the metastases, it is advantageous to image using wide bandwidth filters in order to minimize the exposure time needed, within the dynamic range of the detector. The method detailed here is able to recover multiple sources within an imaging subject simultaneously and so will be applicable to metastasis studies. Additionally, the short timescale of emission of the bioluminescent source and the window of stable emission must be considered. Using wide bandwidth filters with a high transmission is desirable to decrease the data acquisition time.

As shown in this work, using a wide bandwidth filter significantly reduces the quantitative accuracy of BLT, unless the bandwidth is accounted for by using a WBW Jacobian model to reconstruct.
